# Social media as a path to acquire dental home for children: A cross-sectional study in Qatar

**DOI:** 10.5339/qmj.2025.105

**Published:** 2025-12-02

**Authors:** Mohamed A. Hendaus, Mohamed Elkalaf, Eman Bamashmous, Tasneim Osman Elbadri Abdalla, Zahra Hejji, Abdulrahman Al-Debs, Abdelrahman Tayseer Salem, Mohamed Ali, Rasha Abbas Omer, Mohammed Doklaijah, Faisal Siddiqui

**Affiliations:** 1 Qatar University College of Medicine, Doha, Qatar; 2 The View Hospital, in affiliation with Cedars Sinai, Doha, Qatar; 3 Hamad Medical Corporation, Doha, Qatar; 4 Sidra Medicine, Ar-Rayyan, Qatar; 5 University of Michigan, Ann Arbor, MI; 6 University of North Dakota, Grand Forks, ND *Email: mhendaus@yahoo.com

**Keywords:** Pediatrics, oral health, dentistry, dental home, social media

## Abstract

**Background:**

The dental home is essential for maintaining proper oral health in children. This study explores parents’ perspectives on using social media to find a dental home for their preschool-aged children.

**Methods:**

A cross-sectional study was conducted using a questionnaire administered to parents visiting Sidra Medicine, the only tertiary pediatric center in the State of Qatar.

**Results:**

A total of 384 questionnaires were completed by parents and caregivers, resulting in a 94% response rate. The majority of participating parents (66.9%) were male, with over 30 years of age accounting for 45.8%, and about half of them were college graduates. After learning about the concept of a dental home for preschool children, 73% of parents expressed interest. Nearly all participants were active on social media, and about 60.2% preferred to receive oral health messages through these platforms, while 10.9% were unsure. Furthermore, almost 60% believed that social media could influence their decision to seek a dental home, and 77.6% agreed that social media would be an effective way to promote proper oral hygiene. Facebook and Instagram were the two major platforms where our participants preferred to receive information regarding dental health. Importantly, three-quarters of the participants felt that primary care physicians or their representatives should use social media to send reminders about dental home care. Statistical analysis was performed to examine associations between parental age, gender, and educational status in relation to their perceptions of using social media to find a dental home for their children, but the results did not reveal any statistically significant correlations.

**Conclusion:**

Social media can be a valuable tool for disseminating information and promoting the concept of a dental home for preschool children. In addition to social media, pediatricians can play a crucial role in advocating for the idea of a dental home for children.

## 1. INTRODUCTION

Social media consists of computer-mediated technologies that enable the creation and sharing of data, ideas, and various forms of expression among virtual populations and networks.^1^ It thrives on shared content through peer-to-peer (P2P) networks, empowering users to create content rather than just passively consume it. This dynamic interaction has transformed social media into a powerful tool for information and communication, allowing for widespread dissemination.^2^

The surge in social media usage in developing and emerging countries presents a valuable opportunity to enhance health literacy.^3^ It is essential to strategically target audiences based on the platforms they most actively engage with. For instance, a remarkable 80% of individuals in Colombia, Jordan, and Lebanon rely on WhatsApp, whereas only 2% to 4% of the populations in Vietnam and the Philippines utilize the platform.^4^

According to a 2013 online survey, patients are increasingly using social media to gain knowledge, exchange advice, and seek social support. However, they encounter significant barriers due to the unreliability of information and privacy concerns within the patient community.^5^

Tooth decay is a significant health threat to children, affecting their ability to eat, sleep, and function effectively at home and in school. Moreover, untreated dental decay can negatively impact a child’s self-esteem and social development.^6^ Early childhood caries, which affects children aged 71 months or younger, is among the most common preventable diseases worldwide, with a prevalence rate of 1% to 12% in many developed countries.^7,8^

A study from the 2011 Qatar National Oral Health Survey found that an alarming 71.4% of children had dental caries.^9^ Furthermore, another survey revealed an even higher prevalence of caries, at 89.2%, among 4- and 5-year-old Qatari preschool children. This underscores the urgent need for nationwide early preventive measures.^10^

Dental caries in children can cause pain, discomfort, eating disorders, tooth loss, and delayed speech. Furthermore, dental caries affects children’s concentration in school.^11^ In 2009, the total dental expenses for US children aged 5 to 17 years amounted to approximately $20 billion.^12^ To address these challenges, it’s crucial to identify and manage risk factors early, preferably within the first year of an infant’s life, emphasizing the importance of preventing oral diseases. The Dental Home concept is essential for children’s oral health, emphasizing professional counseling and early intervention to prevent dental issues.^13,14^ Establishing a dental home by 12 months ensures children receive necessary preventive care, reducing the risk of dental problems.^15^ While social media is recognized for marketing and patient communication, its role in supporting dental professionals with clinical information is still underexplored.^16^ Additionally, social media’s impact on communication presents challenges for dental educators and their expected roles.^17^

Our research aims to explore parents’ perspectives on using social media to find a dental home for their preschool-aged children. To our knowledge, this is the first study to explore parents’ views on using social media to find a dental home for their preschool children.

### 1.1 Objective

To assess how parents view social media as an important source of information regarding their children’s dental care.

## 2. METHODS

### 2.1 Study type

Our study is a cross-sectional study. To ensure relevancy to our cultural context and diverse education levels, the questionnaire’s questions have been curated from various reputable sources^1–6,11,13,16–21^ and tailored accordingly.

### 2.2 Study location

This study was carried out in the Pediatric Inpatient and Outpatient Departments of Sidra Medicine in Qatar. The Institutional Board Review of Sidra Medicine has permitted us to conduct the study.

### 2.3 Sample size

Due to the absence of similar regional or international studies analyzing the direct relationship between social media influence and dental home, we assumed a correlation factor of approximately 50% and derived our sample size from this hypothesis.

To achieve a 95% confidence level, our initial sample size needed to be 384 participants. Our study did not involve specific interventions and took place from June 1, 2021, to July 1, 2022. We collected data from parents of children aged 1 to 5 years visiting outpatient clinics or hospitalized. Parents filled out questionnaires containing 25 items about demographics, dental health, education, and social media use. There were also open-ended questions for additional comments. Before distributing the questionnaire, we explained its purpose and assured parents that participation was voluntary and their responses would be anonymous. No incentives were offered for participation. The project had approval from the Sidra Medicine Research Office and Institutional Review Board.

### 2.4 Data analysis

We conducted our statistical analysis using the SPSS statistical package, version 21.0 (IBM Corporation, Armonk, NY). A two-sided P-value <0.05 was considered statistically significant. We presented qualitative and quantitative data values as percentages, offering a clear and comprehensive understanding of the data. Our descriptive statistics provided a detailed overview of the demographics and other characteristics of parents and children. Utilizing the chi-square test, we firmly established associations between categorical variables. For small cell frequencies, we employed the chi-square test with a continuity correction factor or Fisher’s exact test. Through univariate and multivariate logistic regression analysis, we effectively assessed the associations of various potential predictors and covariates. It’s important to note that missing data were not accounted for in the analysis, ensuring the integrity and accuracy of our findings.

## 3. RESULTS

Three hundred and eighty-four questionnaires were completed by parents and caregivers of children, resulting in a 94% response rate. Most participating parents (66.9%) were males over 30 years old (45.8%), and half of them were college graduates. Furthermore, 73% of the households had three or fewer children. Table 1 shows the demographic characteristics of the participants.

Upon inquiring about the age at which dental care for children should begin, 182 (47.4%) responded that dental care should start when teeth erupt. This was followed by 91 (23.7%) who said dental care should begin when the child is older than 2 years of age, 49 (12.8%) suggested that dental care should start before tooth eruption, 36 (9.4%) indicated that it should start only if there is a dental issue, and the rest were unsure.

When it comes to preferred methods of oral hygiene, 86 participants (22.4%) mentioned using a combination of brushing teeth and dental floss, followed by 78 participants (20.3%) who only brush their teeth. Fifty-four participants (14.1%) mentioned not brushing at all, and others mentioned using a combination of different methods.

Ninety-nine percent of our participating caregivers use social media, and 303 (78.9%) use it daily. The most common social media platforms are listed in Table 2.

Surprisingly, only 16.1% of parents rated their children’s oral health as excellent, and 137 (35.7%) had not had a dental check-up in the 12 months before the questionnaire. When asked, “How often do you think you need to visit the dentist?” 148 (38.5%) replied “as needed,” followed by 111 (28.9%) saying twice a year, 83 (21.6%) saying once a year, and 38 (9.9%) saying more than twice a year.

After being introduced to the concept of a dental home for preschool children, 283 (73%) of parents expressed interest in acquiring a dental home, while 45 (11.7%) stated no, and the rest were unsure.

Moreover, almost 60% believed that social media could influence their decision to seek a dental home, and 77.6% agreed that social media would be a good way to spread the concept of proper oral hygiene. Table 3 highlights some parental perceptions of the influence of social media on finding a dental home for their children.

It is crucial to highlight that parents aged over 29 demonstrate a significantly higher receptiveness to the concept of a dental home, with a notable P-value of 0.02. This trend is even more pronounced among college graduates, who are far more likely to embrace dental home information delivered through social media, reflecting a striking P-value of 0.0001. Conversely, a deeper analysis reveals that several sociodemographic factors—including parental gender, the number of children, siblings with caries, and employment status—show no statistically significant correlation with receptiveness to dental home concepts (*P* = 0.363, 0.59, 0.220, and 0.079, respectively). While parental age and preferred social media platform come close to significance (*P* = 0.059), the influence of being a female parent cannot be overlooked, exhibiting a compelling significance at *P* = 0.02.

## 4. DISCUSSION

While reviewing the existing literature, we found that no studies specifically assessed the role of social media in helping preschoolers acquire a dental home. This study can serve as a foundation for future research and underscore the significance of social media in enhancing children’s oral health.

This evidence strongly indicates that parental age and education level play pivotal roles in shaping perceptions of dental home concepts. In contrast, factors such as the number of children or siblings with caries and employment status appear to have little impact. This highlights the importance of targeting communications effectively to maximize engagement among parents.

Our research indicates that a large proportion of parents are interested in establishing a dental home for their children, with many preferring to receive oral health messages through social media. Furthermore, many parents believe that social media can influence their decision to seek a dental home and that it could effectively promote the importance of proper oral hygiene. This trend is not surprising, considering that at the start of 2023, Qatar had an internet penetration rate of 99%, with only 1% of the population remaining offline. Out of the total population, 2.62 million people (96.8%) were active social media users, positioning Qatar as one of the countries with the highest social media usage rates in the world.^22^

Krasnova et al.^23^ conducted a study on gender differences in social media usage. Their findings revealed distinct patterns in how young men and women use social media. Men tend to use it primarily for entertainment, while women use it more for communication and obtaining information. The positive attitude toward using social media to find a dental home for preschoolers was not affected by the demographics of our participants.

Social media represents a valuable resource for monitoring mental health. With the vast amount of content generated by users every day, it serves as a rich source of non-intrusive, real-time, and extensive data that can provide valuable insights into individuals’ well-being.^24^ Prominent social networking sites such as Facebook, Twitter, Instagram, Snapchat, LinkedIn, YouTube, and WhatsApp continue to grow in both size and usage.^25^ These platforms are particularly widespread in the Middle East, especially in Qatar.^22^ Utilizing social media to disseminate health information offers several significant advantages over traditional media outlets. It is widely recognized as the fastest channel for sharing alerts and updates about disease outbreaks.^26^ Health institutions can take advantage of social media platforms to share audio podcasts and YouTube videos, thereby enhancing the delivery of health information.^27,28^ Additionally, social media allows for the integration of various media forms to engage the public effectively. For instance, by including hyperlinks, social media posts can direct users to other online resources for more comprehensive health information.^29^ Furthermore, social media serves as an essential communication platform for stakeholders during disease outbreaks. Government social media accounts have become official sources for timely information on disease outbreaks, benefiting local agencies and journalists.^26^

Caregivers play a pivotal role in the pediatric healthcare system. They have a unique opportunity to provide valuable input on the care their children receive and make informed decisions for their loved ones. Their perspectives and understanding of barriers to care may differ significantly from those of healthcare professionals and policymakers.^19^ Understanding parental viewpoints is crucial for developing impactful programs and interventions to reduce barriers and is essential for establishing patient-centered care.^30^

Timely intervention with preventive measures is essential in preventing dental caries in children with special needs. Oral health screening can be seamlessly integrated into routine clinic visits.^19^ According to the American Academy of Pediatrics, conducting the initial dental risk assessment for all children at 6 months of age and establishing a dental home for all children by the age of 12 months is paramount due to the risk of dental caries.^31^

It is concerning to discover that 14% of caregivers in our target population do not prioritize oral hygiene for the children in their care. This indicates a significant lack of community awareness regarding the importance of dental health and the potential consequences of neglecting proper dental care habits. This data emphasizes the urgent need for education and outreach to promote better oral hygiene practices for children.

Poor oral health can have a profound impact on a child’s overall well-being and quality of life. Failing to address this issue promptly can lead to a significant increase in the cost of future dental care.^19^

This study has substantial strengths. The results of our study have both quantitative and qualitative strengths that illustrate the crucial need for dental homes. Our findings will play a pivotal role in shaping more robust programs in Qatar and potentially on a global scale. Nevertheless, we acknowledge the limitations of our study. Certain aspects of parental preference in this context may not have been fully explored. To provide more definitive conclusions, further exploration through larger multicenter studies is imperative. We intend to present our findings to and advocate for the establishment of dental homes for preschool children, utilizing the insights derived from our study.

## 5. CONCLUSION

Social media, as a source of public information, could be an important tool for promoting the concept of a dental home for preschool children. In addition to social media, pediatricians can play a significant role in promoting the idea of a dental home for children.

## CONFLICT OF INTEREST

A preliminary abstract was presented at the American Academy of Pediatrics National Convention and Exhibition in 2021.

## Figures and Tables

**Table 1.** Demographic characteristics of participants.

	*n* (%)
**Parental gender**	
Male	257 (66.9)
Female	127 (33.1)
**Parent age (years)**	
<20	22 (5.7)
20–29	91 (23.7)
30–39	176 (45.8)
>40	94 (24.5)
**Employment status**	
Not working	150 (39)
Working part-time	25 (6.5)
Working full-time	195 (50.8)
Student (full-time)	11 (2.9)
Student (part-time)	3 (0.8)
**Number of children**	
≤3	281 (73.2)
>3	103 (26.8)
**Level of education**	
Less than high school	35 (9.1)
High school	83 (21.6)
College	45 (11.7)
College graduate	177 (46.1)
Higher than a college graduate	43 (11.2)
**Sibling with dental caries**	
Yes	150 (39.1)
No	165 (43.0)
Do not know	58 (15.1)

**Table 2.** Types of social media platforms used.

Type of social media platform	*n* (%)
Facebook + Instagram (± Snapchat)	20 (5.2)
Facebook + YouTube (± Instagram)	40 (10.4)
Facebook + YouTube + Instagram + Snapchat	11 (2.9)
YouTube (± Instagram)	36 (9.4)
YouTube + Instagram + Snapchat	14 (3.6)
Instagram or Snapchat, or both	87 (22.7)
Twitter alone or with other platforms	90 (23.4)
WhatsApp alone or with other platforms	23 (6)
Facebook alone	56 (14.6)

**Table 3.** Some parental perceptions of the influence of social media on finding a dental home for their children.

Questions	Yes, *n* (%)	No, *n* (%)	Do not know, *n* (%)
Do you think social media will influence your decision for a dental home?	221 (57.6)	88 (22.9)	71 (18.5)
Do you think social media needs to increase its focus on dental care?	298 (77.6)	31 (8.1)	52 (13.5)
Do you think there is any difference if the message on social media was sent by a medical party or a commercial dental company?	246 (64.1)	74 (19.3)	60 (15.6)
Do you utilize social media to seek attention for your health?	214 (55.7)	140 (36.5)	27 (7.0)
Do you think social media is good for helping improve the concept of oral hygiene?	298 (77.6)	40 (10.4)	44 (11.5)
Would you like your primary physician to send you reminders on social media regarding dental home care?	280 (72.9)	67 (17.4)	33 (8.6)
Would you like to receive messages on social media regarding oral health?	231 (60.2)	42 (10.9)	111 (28.9)
